# Cellular accumulation and cytotoxic effects of zinc oxide nanoparticles in microalga *Haematococcus pluvialis*

**DOI:** 10.7717/peerj.7582

**Published:** 2019-09-25

**Authors:** Sinouvassane Djearamane, Yang Mooi Lim, Ling Shing Wong, Poh Foong Lee

**Affiliations:** 1 Department of Biomedical Science, Faculty of Science, Universiti Tunku Abdul Rahman, Kampar, Perak, Malaysia; 2 Department of Pre-Clinical Sciences, Faculty of Medicine and Health Sciences, Universiti Tunku Abdul Rahman, Bandar Sungai Long, Selangor, Malaysia; 3 Life Science Division, Faculty of Health and Life Sciences, INTI International University, Nilai, Negeri Sembilan, Malaysia; 4 Department of Mechanical and Material Engineering, Universiti Tunku Abdul Rahman, Bandar Sungai Long, Selangor, Malaysia

**Keywords:** *Haematococcus pluvialis*, Zinc oxide nanoparticles, Algal growth inhibition, Cytotoxicity

## Abstract

**Background:**

Zinc oxide nanoparticles (ZnO NPs) are widely used in household and cosmetic products which imply an increased releasing of these particles into the environment, especially aquatic ecosystems, resulting in the need of assessing the potential toxic effects of ZnO NPS on the aquatic organisms, particularly on microalgae which form the base for food chain of aquatic biota. The present study has investigated the dose- and time-dependent cellular accumulation and the corresponding cytotoxic effects of increasing concentrations of ZnO NPs from 10–200 μg/mL on microalga *Haematococcus pluvialis* at an interval of 24 h for 96 h.

**Methods:**

The scanning electron microscopy-energy dispersive X-ray spectroscopy (SEM-EDX) was used to qualitatively detect the cellular accumulation of ZnO NPs in algal cells, while inductively coupled plasma optical emission spectrometry (ICP OES) was performed to quantify the cell associated-zinc in algal cells. The percentage of cell death, reduction in algal biomass, and loss in photosynthetic pigments were measured to investigate the cytotoxic effects of ZnO NPs on *H. pluvialis*. Extracellular and intracellular changes in algal cells resulted from the treatment of ZnO NPs were demonstrated through optical, scanning, and transmission electron microscopic studies.

**Results:**

SEM-EDX spectrum evidenced the accumulation of ZnO NPs in algal biomass and ICP OES results reported a significant (*p* < 0.05) dose- and time-dependent accumulation of zinc in algal cells from 24 h for all the tested concentrations of ZnO NPs (10–200 μg/mL). Further, the study showed a significant (*p* < 0.05) dose- and time-dependent growth inhibition of *H. pluvialis* from 72 h at 10–200 μg/mL of ZnO NPs. The morphological examinations revealed substantial surface and intracellular damages in algal cells due to the treatment of ZnO NPs.

**Discussion:**

The present study reported the significant cellular accumulation of ZnO NPs in algal cells and the corresponding cytotoxic effects of ZnO NPs on *H. pluvialis* through the considerable reduction in algal cell viability, biomass, and photosynthetic pigments together with surface and intracellular damages.

## Introduction

Zinc oxide nanoparticle (ZnO NP) has been widely used in ceramics, rubber, glass, cement, plastics, lubricants, pigments, paints, skincare, pharmaceutical, food, and textiles industries due to its catalytic, antimicrobial, anti-corrosion, anti-aging, anti-friction, anti-ultraviolet light, and deodorizing properties with large surface for the reactions (Yung et al., 2015; Hou et al., 2018; Moezzi, Mcdonagh & Cortie, 2012; Klingshirn, 2007; Piccinno et al., 2012; Popov et al., 2005). ZnO NP is a transparent and conductive metal oxide nanoparticle that provides clear coatings on transparent surfaces, and at the same time inherits the property of piezoelectricity, which makes it attractive for the manufacturing of electronic products (Ma, Williams & Diamond, 2013). Recently, ZnO NPs have been used in numerous chemotherapeutic drugs such as doxorubicin, 5-flurouracil, and doxorubicin as a carrier (Babu et al., 2017). The estimated global annual production of ZnO NPs as of 2010 was >30,000 metric tons (Keller et al., 2013). The extensive usage of ZnO NPs increases the release of this nanoparticle into the environment and may potentially bring adverse effects to the ecosystem and human health (Ma, Williams & Diamond, 2013). Hence, it is essential to investigate the impact of ZnO NPs on the environment, especially on the aquatic organisms. Previous studies revealed the potential of microalgae as model organisms for the toxicity study of metallic oxide NPs in the environment (Franklin et al., 2007; Griffitt et al., 2008; Navarro et al., 2008; Battin et al., 2009; Manzo et al., 2013; Djearamane et al., 2018). Microalgae have been used as a bio-indicator for freshwater pollutants due to their high bioaccumulation capabilities (Barhoumi & Dewez, 2013) and the ability to indicate the amount of pollutants in water through the changes in algal biomass and photosynthetic pigments (Zhou et al., 2014). The responses of algae to chemical pollutants might be species-specific, thus it is important to conduct tests on different algal species to confirm their responses to certain pollutants (Li et al., 2017).

*Haematococcus pluvialis* is a kind of unicellular, motile, biflagellate, green, and freshwater microalga, with the size of 20–50 μm in diameter and 8–12 μm long (Shah et al., 2016; Kang et al., 2005). *H. pluvialis* grows in freshwater bodies (Burchardt et al., 2006; Klochkova et al., 2013; Chekanov et al., 2014) and it consists of carotenoids (>1.75%), astaxanthin (>1.5%), fatty acids (7–25%), proteins (20–30%), carbohydrates (30–40%), and minerals. It is considered as the best natural source of the commercial product astaxanthin, which is a well-known antioxidant, anticancer, and anti-inflammatory substance (Honga, Choib & Simb, 2016; Matos et al., 2017). In addition, *H. pluvialis* has been used as diets for farmed salmon, trout, sea bream, prawns, and ornamental fish (Dore & Cysewski, 2003). *H. pluvialis* cells are very sensitive to environmental stress and they undergo morphological alterations under various environmental conditions (Hata et al., 2001; Imamoglu, Sukan & Dalay, 2007). Lone et al. (2013) reported that the presence of metallic NPs in the aquatic environments due to several anthropogenic activities might cause detrimental effects on the nutritional quality of the nutrient microalgae through biochemical and physiological alterations. Comotto et al. (2014) revealed the sensitivity of *H. Pluvialis* towards titanium dioxide nanoparticles (TiO_2_ NPs) through a reduction in algal biomass. Hence, the additional information regarding the toxicity effects of ZnO NPs on the nutrient microalga *H. pluvialis* will be useful for the environmental impact assessment of ZnO NPs in the aquatic environment. The information will also help to design methods for screening the contamination of ZnO NPs in microalgae. Otherwise, the intake of ZnO NPs contaminated microalgae may cause health hazards to the consumers. In this paper, cellular accumulation and the corresponding cytotoxic effects of ZnO NPs on freshwater microalga *H. pluvialis* are reported.

## Materials and Methods

### Primary characterization of ZnO NPs

Zinc oxide nanopowder (particle size of >100 nm) was procured from Sigma-Aldrich. The scanning electron microscopy (SEM, S-3400N; HITACHI, Tokyo, Japan) was used to determine the shape and size of ZnO NPs. The chemical composition of the nanomaterial was investigated through scanning electron microscopy energy dispersive X-ray (SEM-EDX) (S-3400N; HITACHI, Tokyo, Japan). Further, X-ray diffractometry (Lab X, XRD-6000; SHIMADZU, Kyoto, Japan) was used to confirm the crystalline structure of ZnO NPs.

### Algal cultivation and exposure to nanoparticles

The freshwater microalga *H. pluvialis* stock culture was obtained from UTEX1926 (University of Texas Culture Collection, Austin, TX, USA). The algal cells were grown in basal bold medium under 17–20 μmol photons/m^2^/s illumination with 16 h light and 8 h dark condition at room temperature (21–23 °C). A stock solution of ZnO NPs (400 μg/mL) was prepared and sonicated for 30 min at 40 kHz to prepare the homogenous suspension of NPs. The exponentially proliferating algal cells with an initial density of 1 × 10^5^ cells/mL were treated with 10, 50, 100, 150 to 200 μg/mL of ZnO NPs, respectively. The cellular accumulation and the toxicity effects of ZnO NPs in algal cells were investigated at an interval of 24 h until 96 h. The experiment included the control algal cells that were free from ZnO NPs treatment.

### Investigation of cellular accumulation of ZnO NPs in algal cells

The algal cells treated with ZnO NPs were centrifuged at 5,000 rpm for 10 min. Then the pelleted cells were washed twice with 1× phosphate-buffered saline (PBS), freeze-dried, and subjected to SEM-EDX (S-3400N; HITACHI, Tokyo, Japan) to detect the cellular accumulation of ZnO NPs in the biomass of *H. pluvialis*. Further, the quantification of ZnO NPs accumulated in the algal cells was performed using inductively coupled plasma optical emission spectroscopy (ICP OES, 5300 DV Perkin Elmer Optima; Akron, Ohio, United States) at 24, 48, 72 and 96 h. Algal cells treated with NPs were pelleted at 5,000 rpm for 10 min and the pelleted cells were washed twice with 1× PBS to remove the loosely bound NPs. Then the algal cells were acid treated with concentrated nitric acid and the zinc content in the algal cell suspension was analyzed using ICP OES.

### Investigation of cytotoxic effects of ZnO NPs on *H. pluvialis*

#### Determination of algal growth inhibition

To determine the cell viability, algal cells treated with ZnO NPs and control cells were loaded in the Neubauer cell counting chamber (Marienfeld, Lauda-Königshofen, Germany). The intact cells without any change in morphology were counted as the viable cells. The effect of NPs on the biomass of *H. pluvialis* was quantified using a spectrophotometer (Genesys 20; Genesys, London, UK) at 680 nm. The photosynthetic pigments of algal cells such as chlorophyll a, carotenoids and astaxanthin were quantified to further confirm the growth inhibitory effects of ZnO NPs on algal cells. The algal cells were washed twice with 1× PBS at 5,000 rpm for 10 min to remove the unbound particles. Subsequently, chlorophyll-a and total carotenoids were extracted in 100% methanol at 65 °C for 60 min or until the cell debris were almost colorless. The extracted pigments were measured using a spectrophotometer (Genesys 20; Genesys, London, UK) at 470, 653, and 666 nm. The equations of Lichtenthaler & Wellburn (1985) were used to quantify chlorophyll-a and carotenoids (Deniz, Saygideger & Karaman, 2011). To quantify astaxanthin, the washed algal cells were treated with 4 N hydrochloric acid at 70 °C for 2 min and cooled. Further, algal cells were subjected to acetone extraction for 1 h and the resultant supernatant was used for quantifying the astaxanthin. The whole extraction process was performed in dim light. The quantity of astaxanthin was determined at 480 nm using an extinction coefficient of 2,500 at 1% level by the method of Davies (Davies, 1976). The percentage of growth inhibition was calculated with respect to the control cells that are devoid of NPs treatment using Eq. (1).

(1) $$I\% = {{\left( {{{\mu }}C - {{\mu}}T} \right)} \over {{{\mu}}C}} \times 100$$

*I*% = percentage of loss in viable cells/biomass/photosynthetic pigments.

μ*C* = mean value of viable cells/biomass/photosynthetic pigments in control.

μ*T* = mean value of viable cells/biomass/photosynthetic pigments in treatment.

#### Microscopic examinations

The optical and scanning electron microscopic examinations were performed to observe the extracellular changes in algal cells. A drop of the sample was placed on a glass slide, sealed with cover glass, and observed under phase-contrast microscope (Nikon, Eclipse, TS 100, Japan). For SEM study, washed algal cells were freeze-dried and subjected for sputtered coating (Sputter Coater SC7620; HITACHI, Tokyo, Japan) and studied through SEM (S-3400N, Scanning Electron Microscope; HITACHI, Tokyo, Japan). The ultrathin sections of algal cells were taken on a copper grid and subjected to transmission electron microscopy (TEM Libra 120; ZEISS, Oberkochen, Germany) to study the intracellular alterations in algal cells.

### Statistical analysis

Statistical analysis was carried out using one-way analysis of variance followed by Tukey’s post hoc test for multiple comparisons (SPSS version 22) at *p* < 0.05. A significant difference at *p* < 0.05 between the control (algal cells without ZnO NPs) and the treatments (algal cells treated with 10, 50, 100, 150, and 200 μg/mL of ZnO NPs) at the specific time period (24, 48, 72, and 96 h) were denoted with the symbol *. All the cytotoxicity tests were conducted in triplicates and the data were presented as mean ± standard deviation.

## Results

### Characterization of ZnO NPs

**Figure 1:**
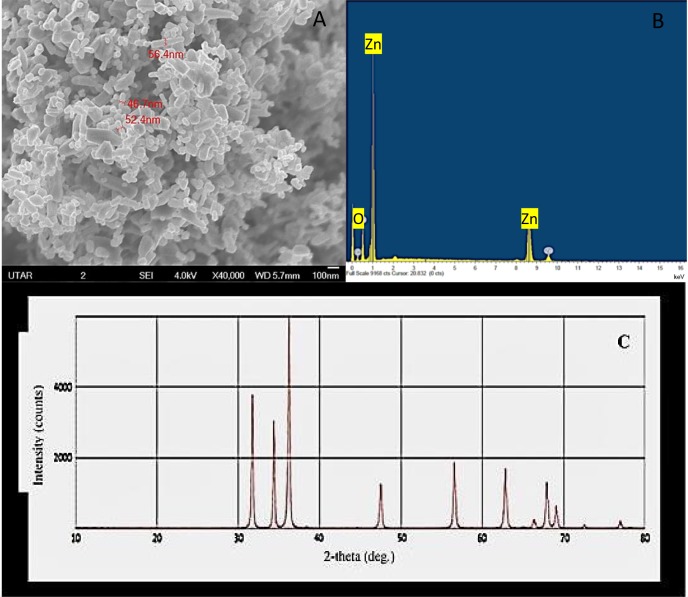
Characterization of ZnO NPs. Scanning electron microscopy image (A), X ray energy dispersive spectroscopy spectrum (B) and X-ray diffractometer spectrum (C) of ZnO NPs.

Scanning electron microscopy micrograph of ZnO nanopowder showed NPs of varied sizes and shapes, such as the sphere, quasi-sphere, hexagon, and rod with agglomerates. The size of the particles was measured to be 51.8 nm with a range of 46.7–56.4 nm (Fig. 1A) and the EDX spectrum of ZnO nanopowder (Fig. 1B) presented peaks corresponded to zinc and oxygen molecules that confirmed the elemental composition of ZnO NPs. Further, the XRD spectrum of the nanopowder (Fig. 1C) displayed strongest diffraction peaks at 31.7°, 34.36°, and 36.19° and also the diffraction peaks at 47.05°, 56.09°, 62.38°, 65.90°, 67.45°, and 68.60°. All diffraction peaks of nanopowder corresponded to the characteristic hexagonal wurtzite crystalline structure of ZnO NPs (Ramesh, Anbuvannan & Viruthagiri, 2015).

### Qualitative analysis of ZnO NPs accumulation in algal biomass

**Figure 2:**
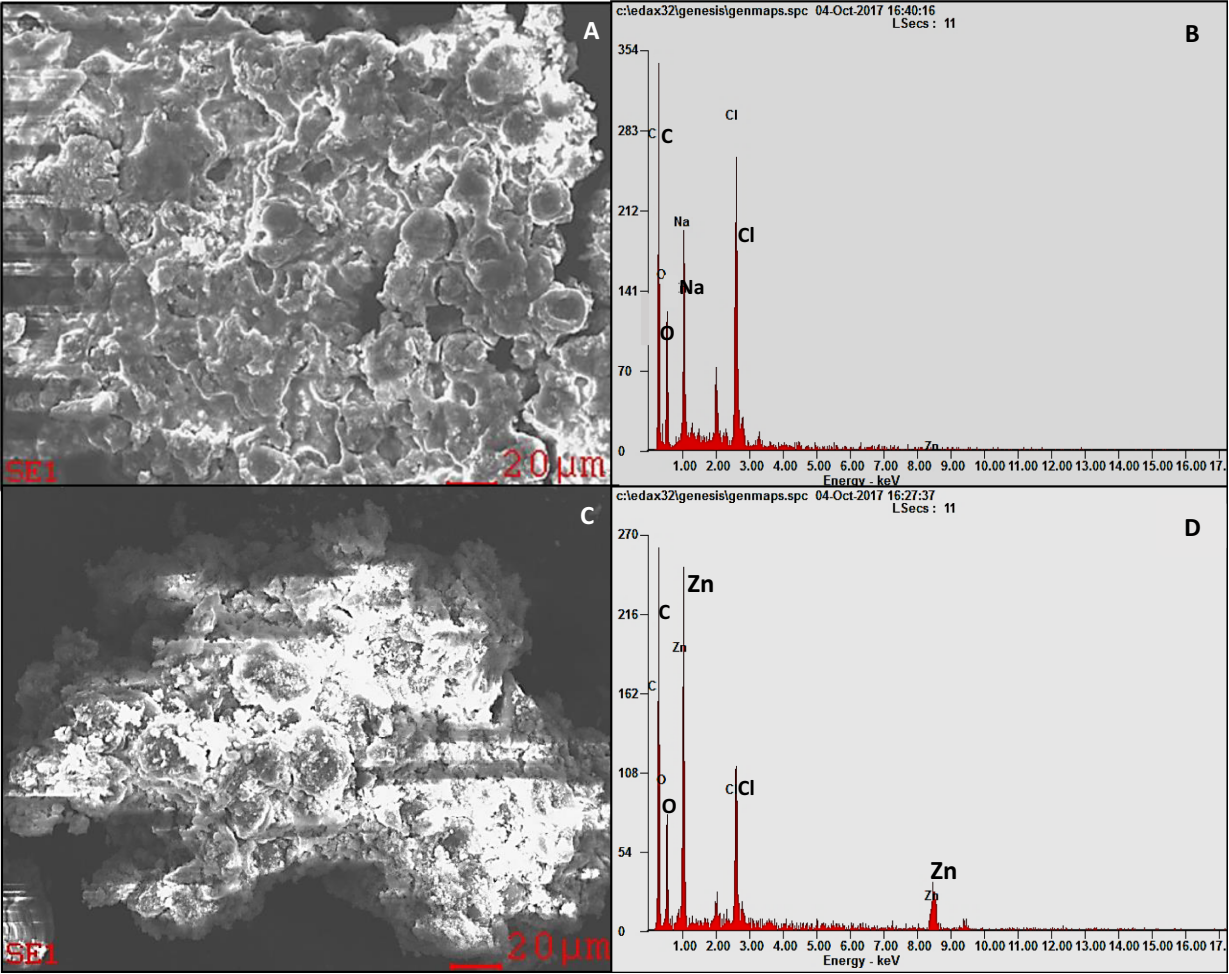
SEM-EDX. SEM micrograph of *H. pluvialis* biomass (A) with the EDX spectrum (B). SEM micrograph of *H. pluvialis* biomass treated with 200 µg/mL of ZnO NPs at 96 h (C) with the EDX spectrum (D).

Scanning electron microscopy image of control algal cells showed smooth spherical non-aggregated cells (Fig. 2A) with no noticeable peak for zinc in the EDX spectrum (Fig. 2B). Whereas, the SEM micrograph of the algal cells treated with ZnO NPs displayed surface accumulation of NPs and the resultant algal cell aggregation (Fig. 2C). The EDX spectral peaks characteristic for zinc were noticed in the algal biomass treated with ZnO NPs (Fig. 2D) that confirmed the accumulation of ZnO NPs in algal biomass.

### Quantification of ZnO NPs accumulation in algal cells

**Figure 3:**
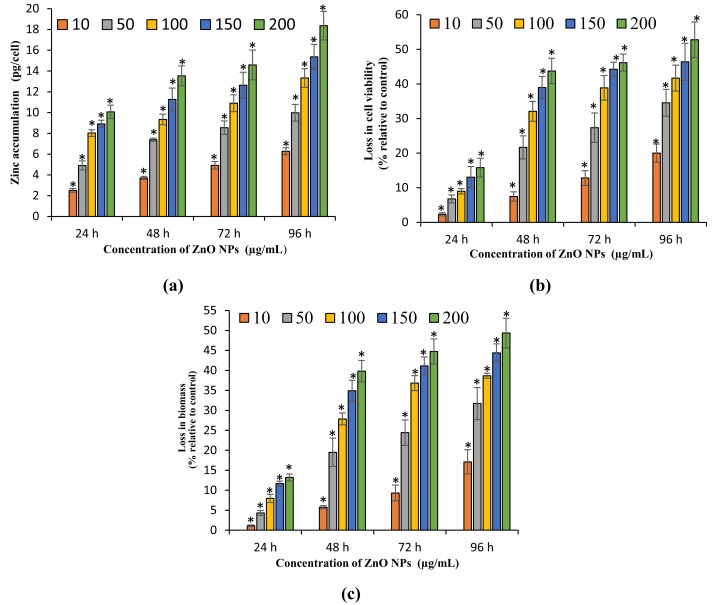
Zinc accumulation, loss in cell viability and biomass. Zinc accumulation in *H. pluvialis* (A). The percentage of loss in cell viability (B) and biomass (C) of *H. pluvialis* upon treatment with ZnO NPs. *, significant difference at *p* < 0.05 between the control and the tested concentrations at the specific time period.

The present study exhibited a dose- and time-dependent accumulation of ZnO NPs in algal cells. The ICP OES results showed a significant (*p* < 0.05) accumulation of zinc in algal cells from 24 h onward for all the tested concentrations of ZnO NPs (10–200 μg/mL). The maximum accumulation of zinc in the algal cells was reported at 96 h with the resultant values of 6.27 ± 0.34, 9.97 ± 0.8, 13.3 ± 0.9, 15.36 ± 1.2, and 18.36 ± 1.38 pg/cell (Fig. 3A) at 10, 50, 100, 150, and 200 mg/L of ZnO NPs, respectively.

### Growth inhibitory effect of ZnO NPs on *H. pluvialis*

**Figure 4:**
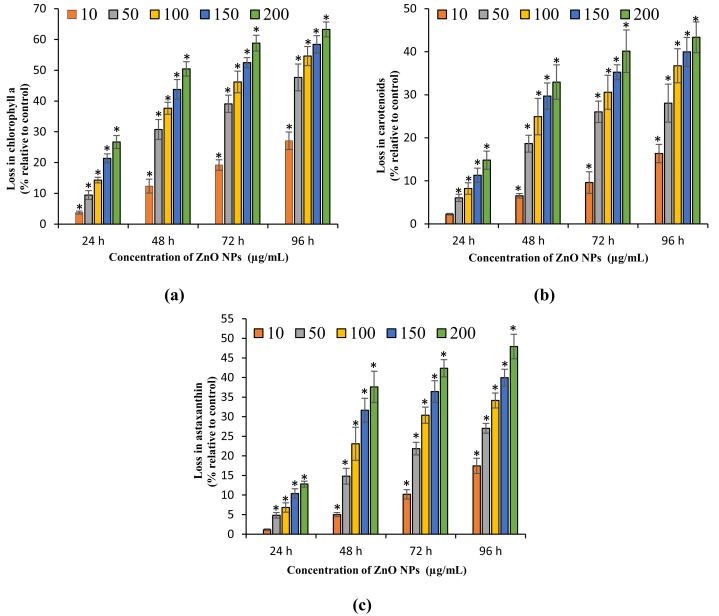
Loss in photosynthetic pigments. Percentage of loss in chlorophyll-a (A), carotenoids (B) and astaxanthin content (C) of *H. pluvialis* upon treatment with ZnO NPs. *, significant difference at *p* < 0.05 between the control and the tested concentrations at the specific time period.

The present study results the growth inhibition of ZnO NPs on *H. pluvialis* reflected by the loss in cell viability, algal biomass, and photosynthetic pigments. The treatment of ZnO NPs caused significant (*p* < 0.05) reduction in cell viability and biomass of *H. pluvialis* from 72 h for all the tested concentrations of ZnO NPs (10–200 μg/mL). The highest growth inhibition was observed at 96 h with 52.78 ± 5.12% loss in cell viability (Fig. 3B) and 49.35 ± 3.69% reduction in algal biomass (Fig. 3C) for 200 μg/mL of ZnO NPs, as compared to control. The growth inhibition of ZnO NPs on *H. pluvialis* was further investigated by measuring the loss in photosynthetic pigments. The results show that the exposure to 10–200 μg/mL ZnO NPs lead to significant loss (*p* < 0.05) in chlorophyll-a at 24 h and carotenoids at 48 h. The maximum reduction in chlorophyll-a and carotenoids were reported to be 63.27 ± 2.4% (Fig. 4A) and 43.35 ± 3.57% (Fig. 4B) respectively, at 200 μg/mL of ZnO NPs for the exposure duration of 96 h. Similarly, the toxicity of ZnO NPs instigated a significant (*p* < 0.05) decline in astaxanthin content from 48 h at 10–200 μg/mL. The maximum reduction in astaxanthin was reported at 96 h with the resultant value of 47.91 ± 3.12% for 200 μg/mL of ZnO NPs (Fig. 4C). The results of the study demonstrated a characteristic time- and dose-dependent growth inhibitory effect of ZnO NPs on *H. pluvialis* through a progressive decrease in cell viability, algal biomass, and the photosynthetic pigments.

### Morphological examination of algal cells treated with ZnO NPs

**Figure 5:**
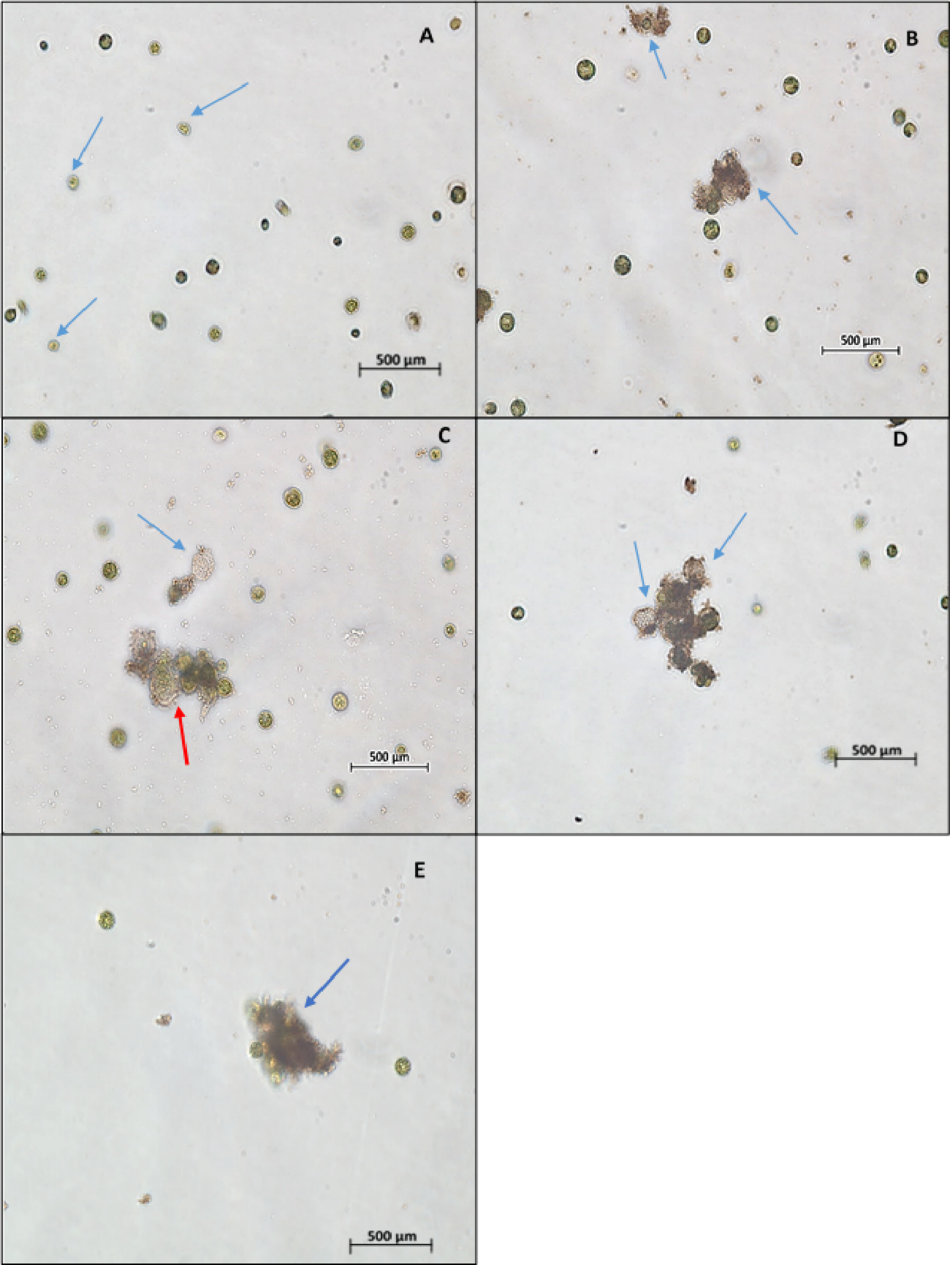
Light microscopy images. Light microscopic image (10×) of control cells shows cell membrane intact cells along with motile cells (blue arrow) (A). Treatment of ZnO NPs on *H. pluvialis* resulted in wrapping of algal cells with NP agglomerates (B), algal cells aggregation, degraded cell (red arrow) and bleached ghost cell (blue arrow) (C), aggregation of algal cells with bleached ghost cells (D) and aggregates of distorted cells (E) with 200 µg/mL of ZnO NPs at 96 h.

**Figure 6:**
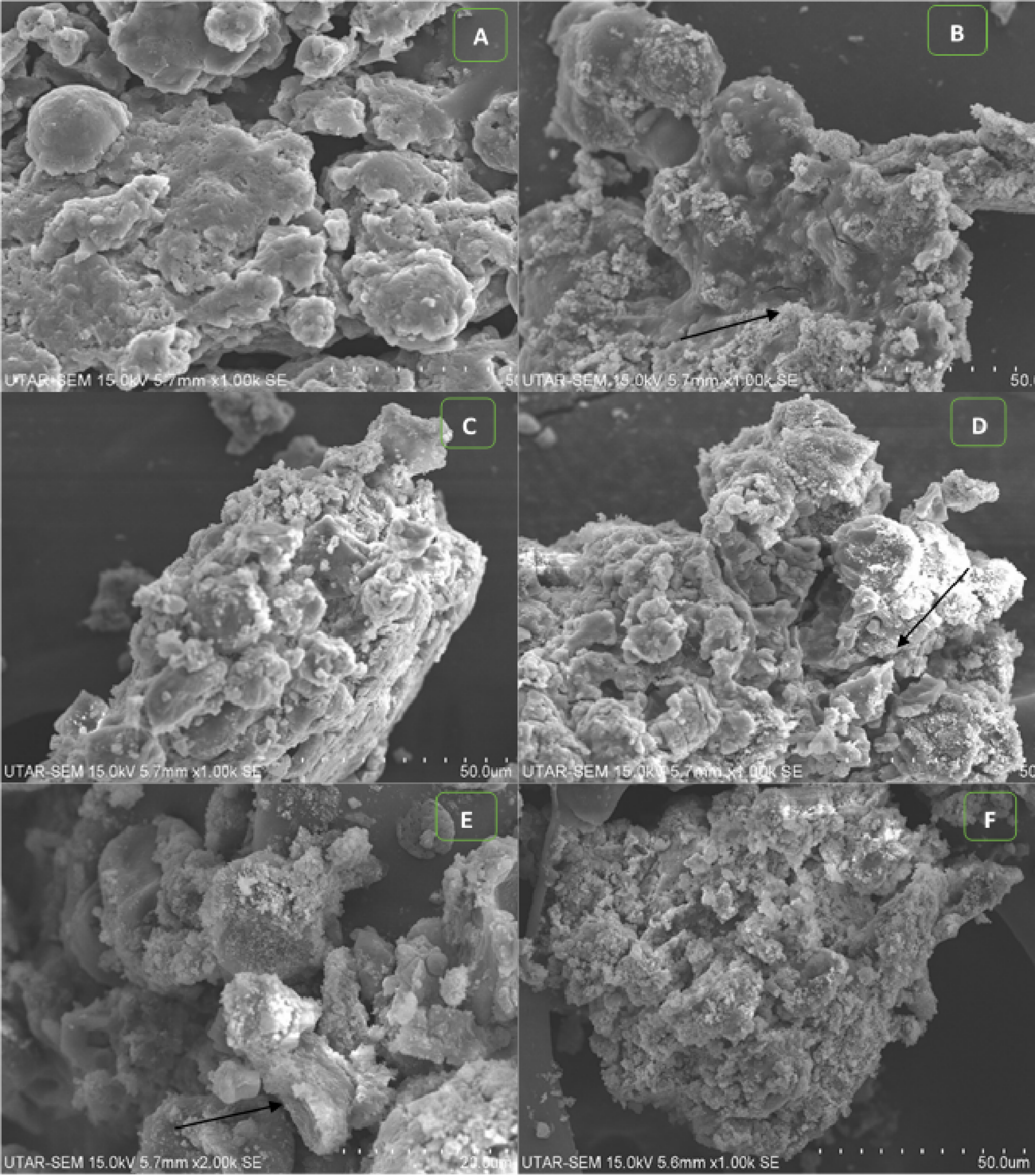
SEM images. SEM micrograph of control cells (A). Treatment of ZnO NPs on *H. pluvialis* resulted in adsorption of NP agglomerates on the algal cells shown by arrowhead (B), aggregates of algal cells (C), distorted cells with altered cell membrane, black arrowhead shows wrinkled cells (D), cell membrane rupture and cell rupture, black arrowhead shows broken cell (E) and aggregation of distorted cells (F) upon treating with 200 µg/mL of ZnO NPs at 96 h. Scale bar—50 µm.

The light microscopic image of algal cells without ZnO NPs treatment (control) showed spherical, membrane-intact cells with numerous motile cells (Fig. 5A). Whereas, the treatment of NPs resulted in adsorption and aggregation of NP agglomerates on algal cells (Fig. 5B), algal cells aggregation, degradation, and bleaching of cells by loss of chlorophyll through cell wall rupture (Fig. 5C), aggregation of algal cells with many ghost cells (Fig. 5D), and clustering of distorted cells (Fig. 5E). Similarly, the SEM image of control cells displayed smooth spherical cells with intact cell membranes (Fig. 6A). On the contrary, ZnO NPs treated algal cells showed entrapment of cells with NP agglomerates (Fig. 6B), algal cells aggregation (Fig. 6C), cell distortion with altered cell membrane (Fig. 6D), cell membrane rupture and the subsequent cell rupture (Fig. 6E), and aggregates of distorted cells (Fig. 6F).

**Figure 7:**
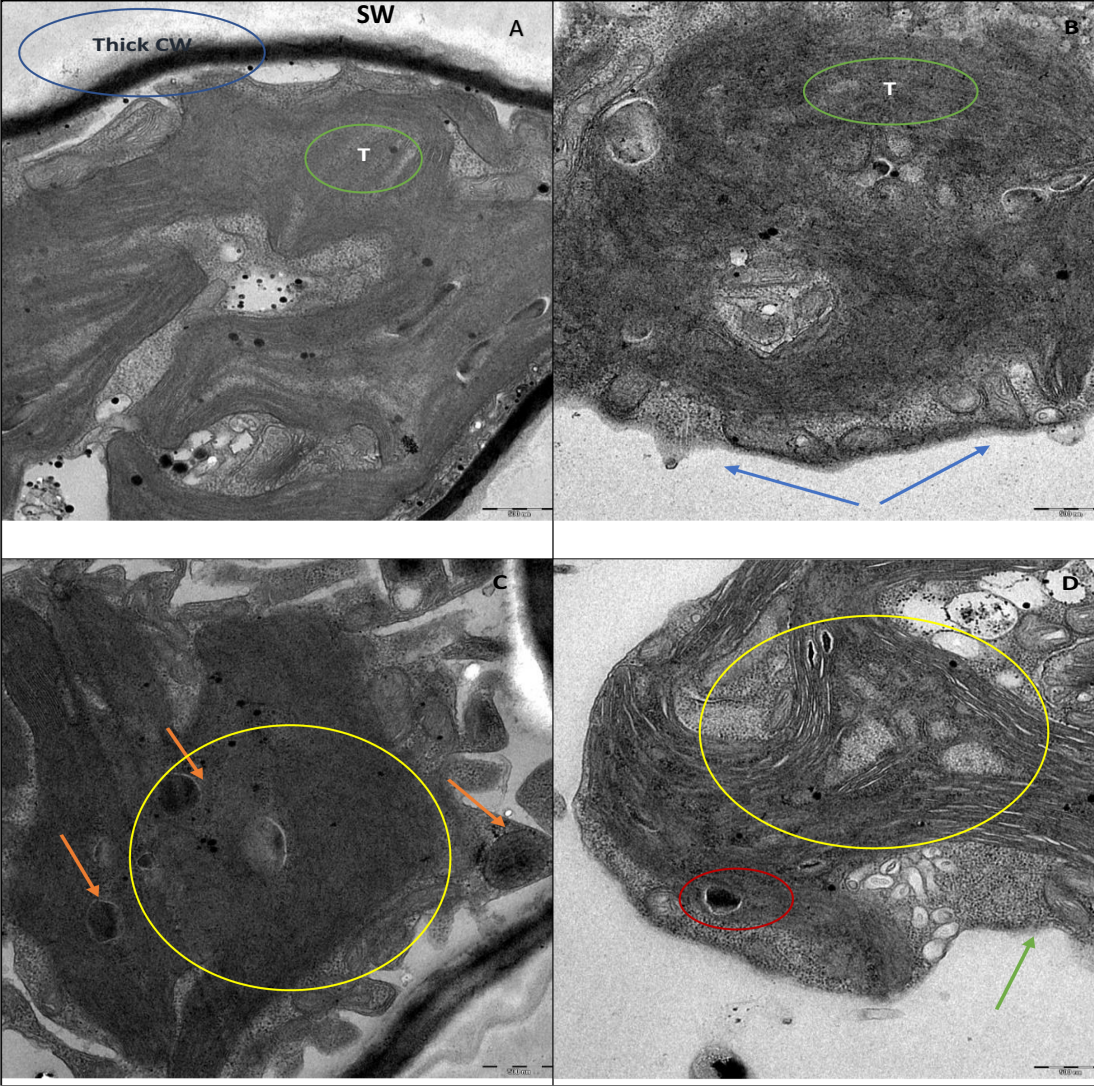
TEM images. TEM micrographs of *H. pluvialis* without ZnO NPs treatment are shown in (A) and (C). TEM picture of control algal cell in (A) displays thick cell wall (CW), smooth secondary wall (SW) and densely packed thylakoids (T). Control cell in (C) shows astaxanthin containing lipid vesicles (orange arrowhead). TEM pictures of *H. pluvialis* treated with ZnO NPs (200 µg/mL) for 96 h are presented in (B) and (D). (B) Displays the complete loss of secondary smooth wall and thick cell wall (blue arrowhead) and destruction of thylakoids (green circle). (D) Shows plasmolysis with leakage of intracellular contents (green arrowhead), shrunken and deformed astaxanthin containing lipid vesicles (red circle in D). The area under the yellow circle in (D) displays the scattered electron transparent cytoplasm resulted from the destruction of intra-cytoplasmic organelles against the electron-dense cytoplasm of the control cell shown in the area under the yellow circle in (C). Scale bar—500 nm.

Furthermore, the TEM micrograph of the control cells shown in Fig. 7A, revealed a thick cell wall surrounding the cell and highly developed chloroplasts with densely packed thylakoids in the cytoplasm. Figure 7C shows the control cell displaying lipid vesicles in the cytoplasm with astaxanthin accumulation. Whereas, the cells treated with ZnO NPs showed complete degradation of thick cell wall together with loss of smooth layers (Fig. 7B). ZnO NPs treatment resulted in plasmolysis with leakage of cytoplasmic contents, deformation of lipid vesicles, and degradation cytoplasmic organelles (Fig. 7D).

## Discussion

Gupta, Sharma & Singh (2014) described two mechanisms in the uptake of metal ions into the algal cells. Firstly, the adsorption of metal ions on the algal surface and the subsequent entry into the cells through cell membranes to get internalized into the cytoplasmic organelles. According to the mechanisms described by Gupta, Sharma & Singh (2014), the results of the present study determined the accumulation of ZnO NPs in the algal biomass through SEM EDX (Figs. 2C and 2D). Similar results were reported by Dmytryk, Saeid & Chojnacka (2014), Zinicovscaia et al. (2016), and Djearamane et al. (2018) who demonstrated the cellular accumulation of selenium, zinc and ZnO NPs in the algal biomass of *Spirulina platensis* through EDX analysis. Furthermore, the quantification of ZnO NPs accumulated in algal cells using ICP OES exhibited a dose- and time-dependent increase in cellular accumulation of ZnO NPs in *H. pluvialis* with highest zinc accumulation of 6.27–18.36 pg/cell (Fig. 3A) for 10–200 mg/L of ZnO NPs at 96 h. Gunawan et al. (2013) demonstrated a dose-dependent cellular uptake of zinc in green alga *Chlamydomonas reinhardtii* with the cellular uptake of zinc ranged from five fg/cell at one mg/L to 18 fg/cell at 100 mg/L on day 8 when exposed to the increasing concentrations of ZnO NPs from one to 100 mg/L. In addition to that, the exposure of duckweed *Spirodela polyrhiza* to 1, 10, and 50 mg/L of ZnO NPs resulted in dose-dependent cellular accumulation of 2.8, 3.6, and 4.5 mg of zinc/g dry weight on day 4 (Hu et al., 2013). A study by Liu et al. (2019) demonstrated the accumulation of silver NPs (Ag NPs) coated with sodium citrate with particle size 20 nm (CIT20) and polyvinylpyrrolidone with particle size 20 nm (PVP20) in zebrafish with the reported values of 30.3 and 66.2 mg/g of silver in intestines, when exposed to 0.61 mg/L of CIT20 and 0.67 mg/L of PVP20 at 96 h respectively. Shen et al. (2013) reported the higher accumulation of zinc in macrophages with 13, 24.6, 99.4 pg/cell compared to monocytes with accumulated zinc of 0.3, 5.1, 22.9 pg/cell at 24 h when treated with 10, 50, and 100 mg/L of ZnO NPs respectively. Similar to the study findings of Shen et al. (2013), the current results revealed a strong relationship between the cellular accumulation of ZnO NPs and cell mortality.

The cytotoxicity parameters of the present study evidently demonstrated a characteristic dose- and time-dependent loss in cell viability and a decrease in algal biomass which confirmed the growth inhibitory effect of ZnO NPs on *H. pluvialis*. A similar trend was observed with the study conducted on freshwater microalga *Chlorella vulgaris* (Suman, Rajasree & Kirubagaran, 2015) and the marine microalga *S. platensis* (Djearamane et al., 2018). However, the present study results reported lower sensitivity of *H. pluvialis* towards ZnO NPs toxicity in comparing with *S. platensis* and *C. vulgaris*. Nevertheless, *H. pluvialis* showed a higher sensitivity to the toxic effects of ZnO NPs when compared with marine alga *S. polyrhiza* (Hu et al., 2013). A study by Yung et al. (2017) confirmed that ZnO NPs had a higher toxicity effect than ZnO and ZnSO_4_ on the marine diatom *Thalassiosira pseudonana*. Numerous earlier studies have reported the sensitivity of *H. pluvialis* to the adverse effects of metals, metallic NPs, and environmental stress. Exposure of *H. pluvialis* to different concentrations of NaCl salt (0.25–2% w/v) for 4 days resulted in 56.6% loss in cell viability and also triggered subsequent steep fall of biomass together with the microscopic evidence of cell lysis at the highest concentration of NaCl (Sarada, Tripathi & Ravishankar, 2002). Li et al. (2008) reported 8.4%, 13.1%, and 19.7% loss in cell viability of *H. pluvialis* at 48 h under various stress conditions when treated with ferrous sulfate (FS), FS + high intensity light (HL) (200 μmol m^−2^ s^−1^) and FS + HL + sodium acetate respectively. A recent investigation by Comotto et al. (2014) reported an 18.1% decrease in biomass of *H. pluvialis* on day 9 with the treatment of 100 μg/mL of TiO_2_ NPs.

In addition, the current results demonstrated a dose- and time-dependent reduction in photosynthetic pigments of *H. pluvialis* corresponding to the loss in cell viability and algal biomass due to the treatment with ZnO NPs. The highest fall in chlorophyll-a, carotenoids, and astaxanthin of *H. pluvialis* were observed at 200 μg/mL on day 4 with the reported values of 63.27%, 43.35%, and 47.91%, respectively (Fig. 4). A similar result was reported by Vidhyavathi et al. (2008) where the stress induced by salt caused 90% and 56.68% reduction in chlorophyll and astaxanthin with a 54.68% decrease in carotenoids of *H. pluvialis* when exposed to 17.1 mM of NaCl on day 9. Besides the salt stress, exposure of *H. pluvialis* to the high intensity of light (97 μmol m^−2^s^−1^) for 48 h resulted in 14.9% and 8.45% decrease in chlorophyll and total carotenoids, respectively (Vidhyavathi, Sarada & Ravishankar, 2009). Numerous studies have described the sensitivity of microalgal pigments toward the toxicity of metals and metallic NPs. Sadiq et al. (2011) showed a dose- and time-dependent loss in the chlorophyll of microalgae *Chlorella* sp. and *Scenedesmus* sp. upon treating with alumina NPs for 72 h. Besides, Oukarroum et al. (2012) demonstrated a concentration-dependent reduction in the chlorophyll of *C. vulgaris* and *Dunaliella tertiolecta* when treated with Ag NPs for 24 h. Sadiq et al. (2011), Djearamane et al. (2019a), (2019b), and Barhoumi & Dewez (2013) have reported the strongest toxic effects of metallic oxide NPs on the electron transport chain of photosynthesis in *C. vulgaris* that resulted in a dose-dependent reduction in the photosynthetic pigment. Unfavorable growth conditions to microalgae such as the stress caused by salt, high light intensity, chemicals, metals, and metal NPs cause reduction in chlorophyll content of microalgae and that could be the major reason for algal growth inhibition as the chlorophyll plays a key role in photosynthesis (Gupta, Sharma & Singh, 2014). Treatment of metals to microalgae results in the destruction of thylakoids that causes a reduction in photosynthetic pigments, which in turn severely affects the photosynthetic activity and causes growth inhibition or cell death (Arunakumara, Zhang & Song, 2008).

Furthermore, the light microscopic images of *H. pluvialis* treated with ZnO NPs revealed wrapping of algal cells with NPs and presence of ghost cells (Fig. 5), in addition to, adsorption of NP agglomerates on algal cells, cell aggregation, alteration in cell structure with wrinkled surface, cell membrane rupture, cell distortion, and cell rupture shown by scanning electron microscopic images (Fig. 6). In accordance with our findings, Comotto et al. (2014) observed the cell aggregation and cell wall degradation in *C. vulgaris*, *H. pluvialis*, and *S. platensis* upon treating with TiO_2_ NPs. Dong et al. (2014) showed strongly wrinkled and damaged cell walls in hydrochloric acid-acetone treated *H. pluvialis* cells. Harker, Tsavalos & Young (1996) demonstrated the fully bleached ghost cells of *H. pluvialis* containing no chlorophyll or carotenoid when exposed to high salt and high light intensity. Such bleaching of cells is thought to happen due to the metabolic imbalance caused by environmental stress and also may be due to the cytoplasmic leakage of cells through cell membrane rupture. In addition, TEM micrographs of ZnO NPs treated *H. pluvialis* showed complete loss of thick cell wall, plasmolysis, destruction of photosynthetic apparatus, degradation and leakage of cytoplasmic contents (Fig. 7). Similar findings were reported by Kasemets et al. (2009) who demonstrated irregularly shaped cells such as shrunken and deformed cells with crushed cell wall and cytoplasmic leakage in yeast *Saccharomyces cerevisiae* upon treating with ZnO NPs. Lee et al. (2013) exhibited phytotoxic effects of ZnO NPs on the medicinal plant *Fagopyrum esculentum* through reduction in biomass of seedling along with damage to surface of root by the aggregates of NPs. Most recently, Hou et al. (2019) demonstrated the genotoxic effects of ZnO NP on zebrafish that the treatment of ZnO NPs affected DNA replication at different phases of cell division which resulted in growth inhibition of zebrafish.

The aggregation and adsorption of NPs on algal surface occur due to the large surface area of NPs and also because of the electrostatic attraction of positively charged ZnO NPs with negatively charged functional groups present in the algal cell wall. The accumulation of nanosized particles on algal cell surface alters cell membrane permeability and causes cell wall damage, that enables excessive entry of NPs into the cells and brings disturbance in the vital functions of internal organelles which subsequently leading to cell death (Kumar et al., 2011; Bhuvaneshwari et al., 2015). Similarly, the algal growth inhibition results from the penetration of NPs into the cell envelope and the following disruption in cell membranes causing cell membrane rupture and leakage of intracellular contents (Chen et al., 2012). The surface binding and the consequent accumulation of nanosized particles on the algal cell surface compromise the cell membrane integrity and cell morphology, and eventually resulting in cell death due to the mechanical damage (Djearamane et al., 2018) or by the intracellular dissociation of metal ions from the internalized NPs that disturbs cellular metabolism (Lin & Xing, 2008). Further, the entrapment of large aggregates of NPs on algal cells reduces the light availability to algal cells (Hazeem et al., 2016) and also the adsorption of nutrients by NPs impairs the availability of nutrients to algal cells, ultimately resulting in growth inhibition (Tang et al., 2013).

## Conclusion

In a nutshell, the treatment of ZnO NPs on *H. pluvialis* resulted in a characteristic dose- and time-dependent accumulation of ZnO NPs in algal cells and triggered the growth inhibitory effects on algal cells together with significant surface and intracellular damages. In addition, the present study demonstrated the following mechanisms that are believed to be responsible for the cytotoxicity effects of ZnO NPs on algal cells; entrapment of algal cells with NPs agglomerates that caused cell membrane damage; physical adsorption and the subsequent uptake of NPs into the algal cells resulted in the destruction of intracellular organelles including photosynthetic apparatus.

## Supplemental Information

10.7717/peerj.7582/supp-1 Supplemental Information 1 Raw data: Loss in cell viability.

10.7717/peerj.7582/supp-2 Supplemental Information 2 Raw data: % loss in biomass.

10.7717/peerj.7582/supp-3 Supplemental Information 3 Raw data: % loss in chlorophyll a.

10.7717/peerj.7582/supp-4 Supplemental Information 4 Raw data: % loss in astaxanthin.

## Additional Information and Declarations

### Data Availability

The following information was supplied regarding data availability: The raw data is available in the Supplemental Files.

## References

[ref-1] Arunakumara K, Zhang X, Song X (2008). Bioaccumulation of Pb^2+^ and its effects on growth, morphology and pigment contents of *Spirulina (Arthrospira) platensis*. Journal of Ocean University of China.

[ref-2] Babu EP, Subastri A, Suyavaran A, Premkumar K, Sujatha V, Aristatile B, Alshammari GM, Dharuman V, Thirunavukkarasu C (2017). Size dependent uptake and hemolytic effect of zinc oxide nanoparticles on erythrocytes and biomedical potential of ZnO-ferulic acid conjugates. Scientific Reports.

[ref-3] Barhoumi L, Dewez D (2013). Toxicity of superparamagnetic iron oxide nanoparticles on green alga *Chlorella vulgaris*. BioMed Research International.

[ref-4] Battin TJ, Kammer FV, Weilhartner A, Ottofuelling S, Hofmann T (2009). Nanostructured TiO_2_: transport behavior and effects on aquatic microbial communities under environmental conditions. Environmental Science & Technology.

[ref-5] Bhuvaneshwari M, Iswarya V, Archanaa S, Madhu G, Kumar GS, Nagarajan R, Chandrasekaran N, Mukherjee A (2015). Cytotoxicity of ZnO NPs towards fresh water algae *Scenedesmus obliquus* at low exposure concentrations in UV-C, visible and dark conditions. Aquatic Toxicology.

[ref-6] Burchardt L, Balcerkiewicz S, Kokocinski M, Samardakiewicz S, Adamski Z (2006). Occurrence of *Haematococcus pluvialis Flotow emend*. Wille in a small artificial pool on the university campus of the collegium biologicum in poznan (Poland). Biodiversity Research and Conservation.

[ref-7] Chekanov K, Lobakova E, Selyakh I, Semenova L, Roman Sidorov R, Solovchenko A (2014). Accumulation of astaxanthin by a new *Haematococcus pluvialis* strain BM1 from the White Sea coastal rocks (Russia). Marine Drugs.

[ref-8] Chen P, Powell BA, Mortimer M, Ke PC (2012). Adaptive interactions between zinc oxide nanoparticles and *Chlorella sp*. Environmental Science & Technology.

[ref-9] Comotto M, Casazza AA, Aliakbarian B, Caratto V, Ferretti M, Perego P (2014). Influence of TiO2 nanoparticles on growth and phenolic compounds production in photosynthetic microorganisms. Scientific World Journal.

[ref-10] Davies B (1976). Carotenoids. Chemistry and biochemistry of plant pigments.

[ref-11] Deniz F, Saygideger S, Karaman S (2011). Response to copper and sodium chloride excess in *spirulina* sp. (cyanobacteria). Bulletin of Environmental Contamination and Toxicology.

[ref-12] Djearamane S, Wong LS, Mooi LY, Lee PF (2018). Cytotoxic effects of zinc oxide nanoparticles on cyanobacterium *Spirulina (Arthrospira) platensis*. PeerJ.

[ref-61] Djearamane S, Wong LS, Mooi LY, Lee PF (2019a). Short-term cytotoxicity of zinc oxide nanoparticles on *Chlorella vulgaris*. Sains Malaysiana.

[ref-62] Djearamane S, Wong LS, Mooi LY, Lee PF (2019b). Cytotoxic effects of zinc oxide nanoparticles on *Chlorella vulgaris*. Pollution Research.

[ref-13] Dmytryk A, Saeid A, Chojnacka K (2014). Biosorption of microelements by *spirulina*: towards technology of mineral feed supplements. Scientific World Journal.

[ref-63] Dong S, Huang Y, Zhang R, Wang S, Liu Y (2014). Four different methods comparison for extraction of astaxanthin from green alga *Haematococcus pluvialis*. The Scientific World Journal.

[ref-14] Dore JE, Cysewski GR (2003). Haematococcus algae meal as a source of natural astaxanthin for aquaculture feeds.

[ref-15] Franklin NM, Rogers NJ, Apte SC, Batley GE, Gadd GE, Casey PS (2007). Comparative toxicity of nanoparticulate ZnO, bulk ZnO, and ZnCl_2_ to a freshwater microalga (*Pseudokirchneriella subcapitata*): the importance of particle solubility. Environmental Science & Technology.

[ref-16] Griffitt RJ, Luo J, Gao J, Bonzongo J-C, Barber DS (2008). Effects of particle composition and species on toxicity of metallic nanomaterials in aquatic organisms. Environmental Toxicology and Chemistry.

[ref-17] Gunawan C, Sirimanoonphan A, Teoh WY, Marquis CP, Amal R (2013). Submicron and nano formulations of titanium dioxide and zinc oxide stimulate unique cellular toxicological responses in the green microalga *Chlamydomonas reinhardtii*. Journal of Hazardous Materials.

[ref-18] Gupta S, Sharma S, Singh S (2014). Hexavalent chromium toxicity to cyanobacterium *Spirulina platensis*. International Research Journal of Pharmacy.

[ref-19] Harker M, Tsavalos AJ, Young AJ (1996). Factors responsible for astaxanthin formation in the chlorophyte *Haematococcus pluvialis*. Bioresource Technology.

[ref-20] Hata N, Ogbonna JC, Hasegawa Y, Taroda H, Tanaka H (2001). Production of astaxanthin by *Haematococcus pluvialis* in a sequential heterotrophic-photoautotrophic culture. Journal of Applied Phycology.

[ref-21] Hazeem LJ, Bououdina M, Rashdan S, Brunet L, Slomianny C, Boukherroub R (2016). Cumulative effect of zinc oxide and titanium oxide nanoparticles on growth and chlorophyll a content of *Picochlorum* sp. Environmental Science and Pollution Research.

[ref-22] Honga M-E, Choib YY, Simb SJ (2016). Effect of red cyst cell inoculation and iron(II) supplementation onautotrophic astaxanthin production by *Haematococcus pluvialis* underoutdoor summer conditions. Journal of Biotechnology.

[ref-23] Hou J, Liu H, Zhang S, Liu X, Hayat T, Alsaedi A, Wang X (2019). Mechanism of toxic effects of Nano-ZnO on cell cycle of zebrafish (*Danio rerio*). Chemosphere.

[ref-24] Hou J, Wu Y, Li X, Wei B, Li S, Wang X (2018). Toxic effects of different types of zinc oxide nanoparticles on algae, plants, invertebrates, vertebrates and microorganisms. Chemosphere.

[ref-25] Hu C, Liu Y, Li X, Li M (2013). Biochemical responses of duckweed (*Spirodela polyrhiza*) to zinc oxide nanoparticles. Archives of Environmental Contamination and Toxicology.

[ref-26] Imamoglu E, Sukan FV, Dalay MC (2007). Effect of different culture media and light intensities on growth of *Haematococcus pluvialis*. International Journal of Natural and Engineering Sciences.

[ref-27] Kang C, Lee J, Park T, Sim SJ (2005). Comparison of heterotrophic and photoautotrophic induction on astaxanthin production by *Haematococcus pluvialis*. Applied Microbiology and Biotechnology.

[ref-28] Kasemets K, Ivask A, Dubourguier H-C, Kahru A (2009). Toxicity of nanoparticles of ZnO, CuO and TiO_2_ to yeast *Saccharomyces cerevisiae*. Toxicology in Vitro.

[ref-64] Keller AA, McFerran S, Lazareva A, Suh S (2013). Global life cycle releases of engineered nanomaterials. Journal of Nanoparticle Research.

[ref-30] Klingshirn C (2007). ZnO: From basics towards applications. Physica Status Solidi (b).

[ref-31] Klochkova TA, Kwak MS, Han JW, Motomura T, Nagasato C, Kim GH (2013). Cold-tolerant strain of *Haematococcus pluvialis* (*Haematococcaceae*, Chlorophyta) from Blomstrandhalvøya (Svalbard). ALGAE.

[ref-32] Kumar A, Pandey AK, Singh SS, Shanker R, Dhawan A (2011). Engineered ZnO and TiO_2_ nanoparticles induce oxidative stress and DNA damage leading to reduced viability of *Escherichia coli*. Free Radical Biology and Medicine.

[ref-33] Lee S, Kim S, Kim S, Lee I (2013). Assessment of phytotoxicity of ZnO NPs on a medicinal plant, *Fagopyrum esculentum*. Environmental Science and Pollution Research.

[ref-34] Li J, Schiavo S, Rametta G, Miglietta ML, La Ferrara V, Wu C, Manzo S (2017). Comparative toxicity of nano ZnO and bulk ZnO towards marine algae *Tetraselmis suecica* and *Phaeodactylum tricornutum*. Environmental Science and Pollution Research.

[ref-35] Li Y, Sommerfeld M, Chen F, Hu Q (2008). Consumption of oxygen by astaxanthin biosynthesis: a protective mechanism against oxidative stress in *Haematococcus pluvialis* (Chlorophyceae). Journal of Plant Physiology.

[ref-36] Lin D, Xing B (2008). Root uptake and phytotoxicity of ZnO nanoparticles. Environmental Science & Technology.

[ref-37] Liu H, Wang X, Wu Y, Hou J, Zhang S, Zhou N, Wang X (2019). Toxicity responses of different organs of zebrafish (*Danio rerio*) to silver nanoparticles with different particle sizes and surface coatings. Environmental Pollution.

[ref-38] Lone J, Kumar A, Kundu S, Lone F, Suseela M (2013). Characterization of tolerance limit in *Spirulina platensis* in relation to nanoparticles. Water, Air, & Soil Pollution.

[ref-39] Ma H, Williams PL, Diamond SA (2013). Ecotoxicity of manufactured ZnO nanoparticles—a review. Environmental Pollution.

[ref-40] Manzo S, Miglietta ML, Rametta G, Buono S, Di Francia G (2013). Toxic effects of ZnO nanoparticles towards marine algae *Dunaliella tertiolecta*. Science of the Total Environment.

[ref-41] Matos J, Cardoso C, Bandarra NM, Afonso C (2017). Microalgae as healthy ingredients for functional food: a review. Food & Function.

[ref-42] Moezzi A, Mcdonagh AM, Cortie MB (2012). Zinc oxide particles: synthesis, properties and applications. Chemical Engineering Journal.

[ref-43] Navarro E, Piccapietra F, Wagner B, Marconi F, Kaegi R, Odzak N, Sigg L, Behra R (2008). Toxicity of silver nanoparticles to *Chlamydomonas reinhardtii*. Environmental Science & Technology.

[ref-44] Oukarroum A, Bras S, Perreault F, Popovic R (2012). Inhibitory effects of silver nanoparticles in two green algae, *Chlorella vulgaris* and *Dunaliella tertiolecta*. Ecotoxicology and Environmental Safety.

[ref-45] Piccinno F, Gottschalk F, Seeger S, Nowack B (2012). Industrial production quantities and uses of ten engineered nanomaterials in Europe and the world. Journal of Nanoparticle Research.

[ref-46] Popov AP, Priezzhev AV, Lademann J, Myllylä R (2005). TiO_2_ nanoparticles as an effective UV-B radiation skin-protective compound in sunscreens. Journal of Physics D: Applied Physics.

[ref-47] Ramesh AM, Anbuvannan MB, Viruthagiri GB (2015). Green synthesis of ZnO nanoparticles using *Solanum nigrum* leaf extract and their antibacterial activity. Spectrochimica Acta Part A: Molecular and Biomolecular Spectroscopy.

[ref-48] Sadiq IM, Pakrashi S, Chandrasekaran N, Mukherjee A (2011). Studies on toxicity of aluminum oxide (Al_2_O_3_) nanoparticles to microalgae species: *Scenedesmus* sp. and *Chlorella* sp. Journal of Nanoparticle Research.

[ref-49] Sarada R, Tripathi U, Ravishankar G (2002). Influence of stress on astaxanthin production in *Haematococcus pluvialis* grown under different culture conditions. Process Biochemistry.

[ref-50] Shah M, Mahfuzur R, Liang Y, Cheng JJ, Daroch M (2016). Astaxanthin-producing green microalga *Haematococcus pluvialis*: from single cell to high value commercial products. Frontiers in Plant Science.

[ref-51] Shen C, James SA, De Jonge MD, Turney TW, Wright PFA, Feltis BN (2013). Relating cytotoxicity, zinc ions, and reactive oxygen in ZnO nanoparticle-exposed human immune cells. Toxicological Sciences.

[ref-52] Suman TY, Rajasree SRR, Kirubagaran R (2015). Evaluation of zinc oxide nanoparticles toxicity on marine algae *Chlorella vulgaris* through flow cytometric, cytotoxicity and oxidative stress analysis. Ecotoxicology and Environmental Safety.

[ref-53] Tang Y, Li S, Qiao J, Wang H, Li L (2013). Synergistic effects of nano-sized titanium dioxide and zinc on the photosynthetic capacity and survival of *Anabaena* sp. International Journal of Molecular Sciences.

[ref-55] Vidhyavathi R, Sarada R, Ravishankar GA (2009). Expression of carotenogenic genes and carotenoid production in *Haematococcus pluvialis* under the influence of carotenoid and fatty acid synthesis inhibitors. Enzyme and Microbial Technology.

[ref-56] Vidhyavathi R, Venkatachalam L, Sarada R, Ravishankar GA (2008). Regulation of carotenoid biosynthetic genes expression and carotenoid accumulation in the green alga *Haematococcus pluvialis* under nutrient stress conditions. Journal of Experimental Botany.

[ref-57] Yung MMN, Kwok KWH, Djurišić AB, Giesy JP, Leung KMY (2017). Influences of temperature and salinity on physicochemical properties and toxicity of zinc oxide nanoparticles to the marine diatom *Thalassiosira pseudonana*. Scientific Reports.

[ref-58] Yung MMN, Wong SWY, Kwok KWH, Liu FZ, Leung YH, Chan WT, Li XY, Djurišić AB, Leung KMY (2015). Salinity-dependent toxicities of zinc oxide nanoparticles to the marine diatom *Thalassiosira pseudonana*. Aquatic Toxicology.

[ref-59] Zhou H, Wang X, Zhou Y, Yao H, Ahmad F (2014). Evaluation of the toxicity of ZnO nanoparticles to *Chlorella vulgaris* by use of the chiral perturbation approach. Analytical and Bioanalytical Chemistry.

[ref-60] Zinicovscaia I, Chiriac T, Cepoi L, Rudi L, Culicov O, Frontasyeva M, Rudic V (2016). Selenium uptake and assessment of the biochemical changes in *Arthrospira (Spirulina) platensis* biomass during the synthesis of selenium nanoparticles. Canadian Journal of Microbiology.

